# Misalignment of the Desired and Measured Center of Pressure Describes Falls Caused by Slip during Turning

**DOI:** 10.1371/journal.pone.0155418

**Published:** 2016-05-11

**Authors:** Takeshi Yamaguchi, Hironari Higuchi, Hiroshi Onodera, Kazuo Hokkirigawa, Kei Masani

**Affiliations:** 1 Graduate School of Engineering, Tohoku University, Sendai, Miyagi, Japan; 2 Graduate School of Biomedical Engineering, Tohoku University, Sendai, Miyagi, Japan; 3 Photon Science Center and Department of Electronic Engineering, School of Engineering, The University of Tokyo, Bukyo-ku, Tokyo, Japan; 4 Rehabilitation Engineering Laboratory, Institute of Biomaterials and Biomedical Engineering, University of Toronto, Toronto, Ontario, Canada; 5 Rehabilitation Engineering Laboratory, Lyndhurst Centre, Toronto Rehabilitation Institute – University Health Network, Toronto, Ontario, Canada; Universidad Europea de Madrid, SPAIN

## Abstract

In this study, desired center of pressure (dCOP) was introduced to evaluate dynamic postural stability. The dCOP is defined as a virtual point on the ground, where the moment around the body center of mass (COM) becomes zero when dCOP and the measured COP (mCOP) coincide. We hypothesized that, when the misalignment of the dCOP and mCOP (dCOP-mCOP) increases up to a certain value due to a large perturbation during walking, it becomes difficult to make a compensatory step and to recover balance of COM and to continue gait. Here we tested this hypothesis in slipping during turning. The study involved twelve healthy young adult males with an average age of 21.5±1.9 yrs. The subjects were asked to (1) walk straight and turn 60 degrees to the right with the right foot (spin turn) on a dry floor surface, and (2) walk straight and 60 degrees spin turn to the right on a slippery lubricated surface. The dCOP-mCOP during turning in the slip trial with fall were significantly larger, particularly in *x*-direction (i.e., the medial-lateral direction during straight walk), than that in no-slip trial and slip trial without fall. The receiver operating characteristic (ROC) analysis indicated that the dCOP-mCOP in *x*-direction is good indicator of falling (area under the curve (AUC) = 0.93) and the threshold in the dCOP-mCOP in *x*-direction to distinguish for fall or no-fall was 0.55 m. These results support our hypothesis in slipping during turning.

## Introduction

The dynamic postural stability of the human gait has been attracting a lot of attention from many researchers. The majority of such biomechanical studies have focused on the relationship between the center of mass of the entire body (COM) and base of support (BOS) defined by the feet. That is, to maintain postural stability, the displacement and the velocity of the COM must be regulated within the stability limits of the BOS [1–5]. Measures for assessing the dynamic postural stability have been proposed on the basis of the relationship between the COM and the BOS limit using an inverted pendulum model. Pai and Patton [1] developed a feasible stability region (FSR) to show all feasible COM motion state, i.e., combinations of horizontal COM velocities and positions, which allows a successful termination of the COM motion over the BOS. The instantaneous COM stability, which is the distance from a given state of the COM motion to the limits of FSR, has been used as a measure of dynamic postural stability [4,6]. Hof, et al. [2] proposed an extrapolated center of mass (XCoM), which is defined as the COM position with an addition of a linear function of the COM velocity and which must be positioned over the BOS to maintain postural balance during steady state walking. The margin of stability, which is the distance between the XCoM and the border of the BOS [2, 7], has recently been widely used as a measure of dynamic postural stability during steady state walking [8–10].

When a large balance perturbation exists, such as slips, trips, and floor perturbation, any kind of stabilizing reactions, such as compensatory steps, are required, which alter the BOS to avoid fall and to continue or successfully terminate gait [11]. The majority of researches on gait responses following a large perturbation focused on the nature of recovery responses, quantifying joint torques, and/or the muscle activity, whereas they did not propose any measures of dynamic postural stability [12–14]. In case of XCoM concept, once XCoM passes beyond the BOS limit due to large perturbations, the margin of stability indicates the person’s posture is unstable and requires compensatory reactions but do not indicate how much unstable the posture is [13]. The instantaneous COM stability is a measure that can quantify the amount of instability and indeed can identify fall and recovery from forward slip during straight walking [6]. However, there is a practical difficulty in using this in a complex and realistic situation such as slipping during turning walking.

Turning requires higher friction between shoe and floor to prevent slipping compared with straight walking, resulting in being more slippery than straight walking [15, 16]. A slip is likely to occur in the mediolateral direction during turning, which needs complicated compensatory stepping such as cross-over stepping and results in the increased risk of fall [17]. The COM is prone to locate outside the BOS for nearly the entire stance phase during spin turns, i.e., turn to right (left) with a right (left) foot [18, 19]; thus it becomes unstable when slip occurs during spin turn compared with a straight gait [17, 19]. To our best knowledge, the dynamic postural stability evaluation with slip during turning has not been studied and only few measures have been proposed which could be applicable to assessing the dynamic postural stability under large perturbations such as slipping during turning [20, 21].

In this study, we introduce a desired center of pressure (dCOP) that yields a new measure to assess the dynamic postural stability in recovering balance from slipping during turning. The nomenclature of “desired center of pressure” was to indicate the desired COP location where the moment around the COM does not occur. The location of dCOP is equivalent to “target zero moment point (ZMP)” [22] or “centroidal moment pivot (CMP)” [23] used in Robotics. Although human body does not take the same strategy as the one used by these robots, this concept can be used to assess the dynamic postural stability during walking. As shown in the following section, we hypothesize that, when the distance between dCOP and the measured center of pressure (mCOP) increases up to a certain value due to a large perturbation, it becomes difficult to recover balance of COM and to continue gait. We aimed to test this hypothesis in slipping during turning. In this study, a spin turn task during walking on slippery floor was applied to induce slip and to threaten the dynamic postural stability.

## Methods

### Desired center of pressure (dCOP)

**Fig 1:**
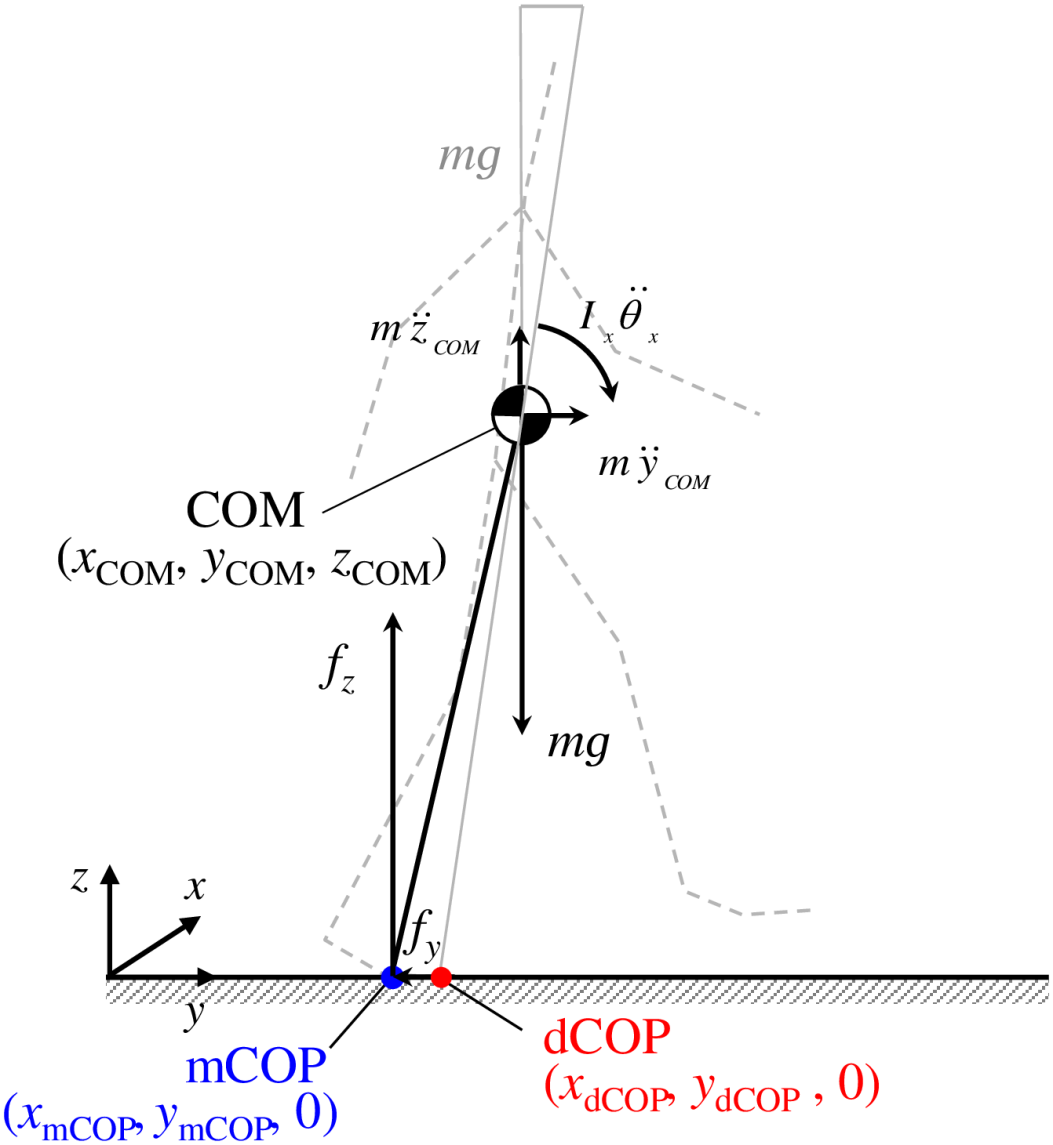
Inverted pendulum model and the desired center of pressure (dCOP) in sagittal plane. The dCOP is defined as a virtual point on the ground, i.e., location where the moment around the body center of mass (COM) becomes zero when dCOP and the measured COP (mCOP) are coincident.

The dCOP is defined as a virtual point on the ground, which is calculated based on the kinematics of the COM. We assume an inverted pendulum model with all mass at the COM location with a reaction force applied at the actual, global center of pressure of supporting foot (or feet when double support period) measured using a force plate (mCOP) (Fig 1). When we consider *y*-*z* plane (sagittal plane with *y* and *z* standing for the horizontal and vertical axes, respectively), the equations of motion are:

$$m{\ddot{y}}_{COM}=f_{y}$$ (1)

$$m{\ddot{z}}_{COM}=f_{z}-m\text{g}$$ (2)

$$I_{x}{\ddot{\theta }}_{x}=f_{z}(y_{COM}-y_{mCOP})-f_{y}z_{COM}$$ (3)

where *f*_y_ and *f*_z_ are horizontal and vertical ground reaction force; *m* and *g* are the whole body mass and the gravitational acceleration; *y*_*COM*_ and *z*_*COM*_ are *y* and *z* coordinates of the COM position; ${\ddot{y}}_{COM}$ and ${\ddot{z}}_{COM}$ are the horizontal and vertical acceleration of the COM; *y*_mCOP_ is the *y* coordinates of the mCOP; *I*_*x*_ and ${\ddot{\theta }}_{x}$ are the moment of inertia of the whole body (assumed rigid) and angular acceleration around the whole-body COM in *y*-*z* plane. The mCOP position is given by combining Eqs (1) and (2) with Eq (3):

$$y_{mCOP}=y_{COM}-\frac{{\ddot{y}}_{COM}}{{\ddot{z}}_{COM}+g}z_{COM}-\frac{I_{x}{\ddot{\theta }}_{x}}{m({\ddot{z}}_{COM}+g)}$$ (4)

When the rotation of the pendulum is avoided (i.e. the moment about the COM is not applied), the angular acceleration of the COM in Eq (4) (i.e., the third term on the right side) becomes zero. The center of pressure (COP) position under this non-moment condition is defined as the dCOP, for the y-z plane (sagittal plane), the *y* coordinate of which is described as:

$$y_{dCOP}=y_{COM}-\frac{{\ddot{y}}_{COM}}{{\ddot{z}}_{COM}+g}z_{COM}$$ (5)

In the same manner for the *x*-*z* plane (frontal plane), the *x* coordinate of the dCOP (*x*_dCOP_) is described as:

$$x_{dCOP}=x_{COM}-\frac{{\ddot{x}}_{COM}}{{\ddot{z}}_{COM}+g}z_{COM}$$ (6)

where *x*_*COM*_ and ${\ddot{x}}_{COM}$ are *x* coordinate of the COM position and the horizontal acceleration of the COM, respectively. Eqs (5) and (6) can be written using ground reaction force components as:

$$y_{dCOP}=y_{COM}-\frac{f_{y}}{f_{z}}z_{\text{COM}}$$ (5’)

$$x_{dCOP}=x_{COM}-\frac{f_{\text{x}}}{f_{\text{z}}}z_{\text{COM}}$$ (6’)

However, we did not use these equations in this manuscript, as we were not able to completely rely on the load cell force due to its single axis configuration.

The moment around COM in *y*-*z* and *x*-*z* planes is described using dCOP and mCOP as follows:

$$I_{x}{\ddot{\theta }}_{x}=m(y_{dCOP}-y_{mCOP})({\ddot{z}}_{COM}+g)\;(\text{in }y-z\text{ plane})$$ (7)

$$I_{y}{\ddot{\theta }}_{y}=m(x_{dCOP}-x_{mCOP})({\ddot{z}}_{COM}+g)\;(\text{in }x-z\text{ plane})$$ (8)

where *I*_*y*_ and ${\ddot{\theta }}_{y}$ are the moment of inertia of the whole body and the angular acceleration around the whole-body COM in *x*-*z* plane. According to Eqs (7) and (8), the moment around COM increases with an increase in the misalignment between dCOP and mCOP (dCOP-mCOP) and/or $m({\ddot{z}}_{COM}+g)$ that is identical to the vertical ground reaction force *f*_z_ (see Eq (2)). The resultant *f*_z_ varies approximately from 70% to 130% of body weight during walking, while dCOP-mCOP can vary much wider. For example, we calculated the resultant *f*_z_ values and dCOP-mCOP values during a stance phase in straight walking on a dry level floor with a natural pace (young adult male), and the *f*_z_ varied from 448N to 741N (75% to 123% of body weight) while the dCOP-mCOP varied from 0.013 m to 0.206 m. The maximum/minimum values for *f*_z_ and dCOP-mCOP are 1.6 and 16, respectively. We also compared the variation of dCOP-mCOP with that of *f*_z_ during stance phase in 60° spin turn for a young adult male with his natural speed. The results were approximately equivalent to those of straight walking. That is, the *f*_z_ varied from 473 N to 692 N (79% to 116% of body weight) while the dCOP-mCOP varied from 0.002 m to 0.248 m; the maximum/minimum values for *f*_z_ and dCOP-mCOP are 1.5 and 100, respectively. Thus, dCOP-mCOP must dominate the moment around the COM, and can be utilized as a measure of the dynamic postural stability during gait. In addition, the length of compensatory step increases when the dCOP-mCOP increases due to a large perturbation, which implies that the dCOP location suggest the desired foot placement to maintain dynamic postural stability. As dCOP-mCOP does not require to be considered with BOS, dCOP-mCOP can be applicable under large perturbation conditions with compensatory steps. Thus, we hypothesize that, when dCOP-mCOP increases up to a certain value due to a large perturbation during walking, it becomes difficult to recover balance of COM and to continue gait.

### Subjects

The study involved twelve healthy young adult males with no neuromusculoskeletal disorders (age 21.5 ± 1.9 years; height 1.70 ± 0.05 m; mass 63.5 ± 5.9 kg). This study was approved by the Institutional Review Board of National Nishitaga Hospital. Each participant provided written informed consent to participate in this study.

### Experimental procedure

**Fig 2:**
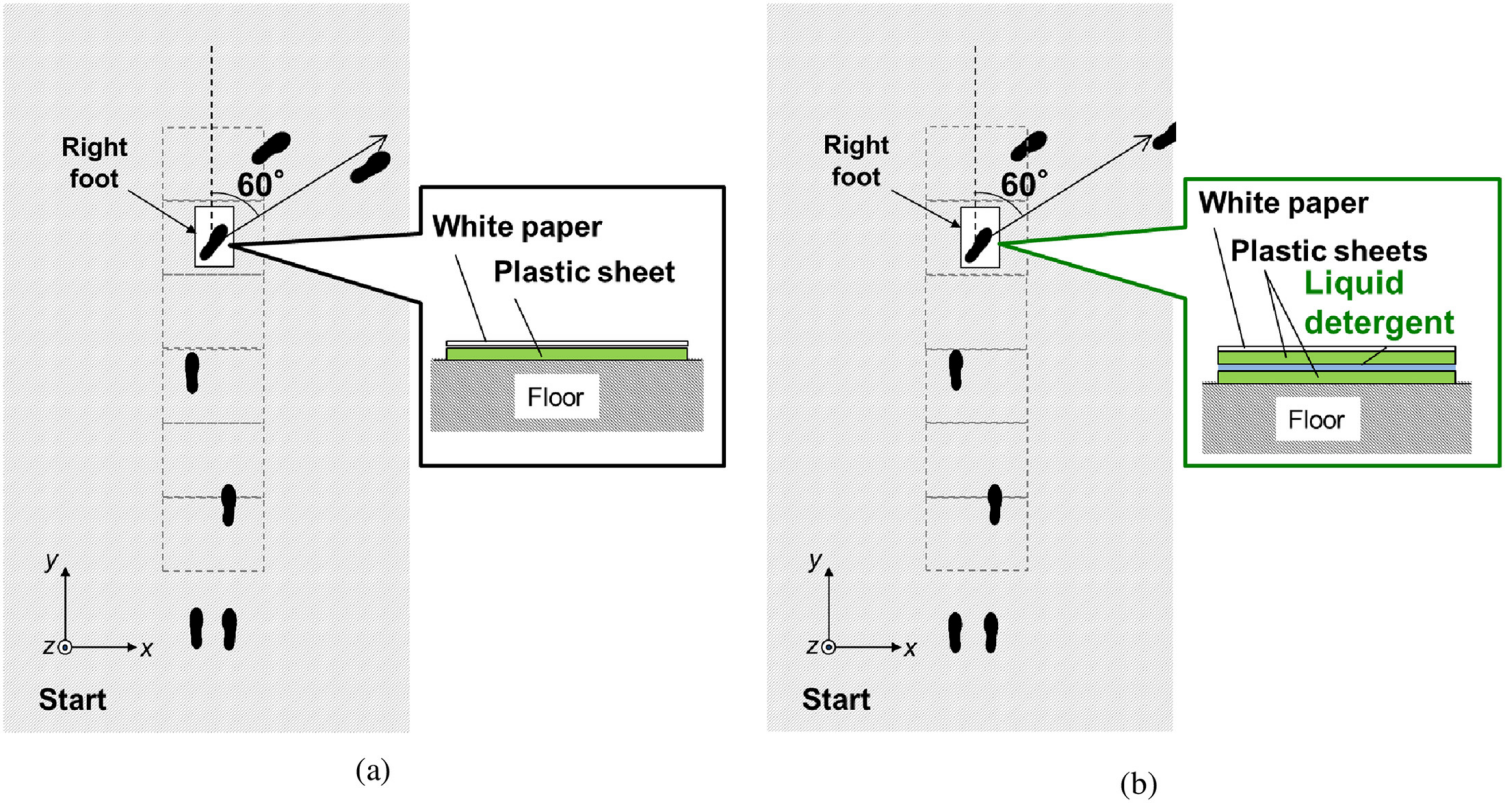
Experimental set-up and movement instruction: (a) straight walk and 60 degrees spin turn on the dummy sheet and (b) straight walk and 60 degrees spin turn on the slip sheet.

Fig 2 shows schematic diagrams of the experimental set-up and instructions given to subjects. *x*- and *y*-coordinate respectively was set as the anteroposterior and mediolateral directions during straight walk. Six force plates (2×model 9807; 4×model 9260AA6, Kislter Corporation, Winterthur, Switzerland) were installed in series within a 5-m walkway. An eight-camera motion measurement system (MAC 3D System, Motion Analysis Corporation, California, USA) measured full body kinematics from 25 reflective markers placed bilaterally to all four extremities, head, and the torso, where markers on lower extremity were placed based on Helen Hayes marker set [24]. All data were collected with a sampling frequency of 120 Hz. The subjects were provided a pair of commercially available walking shoes with a rubber (NBR) sole to eliminate the effect of the difference in shoe sole materials and tread grooves on slip resistance.

The subjects were asked to walk straight for 2 steps, and then turn 60 degrees to the right with the right foot on the sheet placed on the 5^th^ force plate. The degree of turning (60 degrees) was selected based on the previous research [17]. There were two types of sheets; one is the dummy sheet [A4 white paper was adhered to 0.1-mm-thick polyester sheet, Fig 2(a)] and the other is the slip sheet [the polyester sheets between which liquid detergent (viscosity: 140 mPa·s) was trapped and the A4 white paper was adhered to the top polyester sheet, Fig 2(b)]. Each subject performed seven trials: two dummy-sheet-trials (without slip) and five slip-sheet-trials (with slip trials). The order of the dummy-sheet-trial and slip-sheet-trial was randomized to eliminate learning effect. Subjects did not have any prior knowledge of number of trials for each condition, were neither told nor were able to visually identify the type of sheet. They were asked to walk as naturally as possible at a self-selected pace. The subjects were allowed to step after slip to recover their balance and continue walking in the turning direction. During the practice period, the starting position was adjusted to ensure that the foot strike was on the sheet with their third step with subject’s natural step length. Subjects wore a safety harness that was designed to prevent impact with the floor without otherwise restricting gait and movement that would help in maintaining balance. A load cell mounted between the harness and a guide rail (LCS15T001, A&D Company, Limited, Tokyo, Japan) was used to measure the force exerted on the subject during turning. All trials were videotaped.

### Data analysis

The whole body COM behavior (position, velocity and acceleration) was estimated using a thirteen-segment model from kinematic data with a motion analysis software (Visual 3D, C-Motion Inc., Maryland, USA). The global COP location, i.e. mCOP, was also computed from the ground reaction force data with the motion analysis software. The subsequent analyses were conducted using Matlab (Mathworks Inc., Massachusetts, USA). The ground reaction force and kinematics data were low-pass filtered with a cut-off frequency of 6 Hz using a fourth-order, zero-lag, Butterworth filter. The dCOP location (*x*_dCOP_, *y*_dCOP_, 0) was computed from the COM kinematic data using Eqs (5) and (6), with which dCOP-mCOP was also computed. A fall was identified when the peak force exerted on the load-cell exceeded 30% of the subject's body weight because of slipping, and it was confirmed via visual inspection of recorded video [25]. The trials were categorized into three groups based on this fall identification, i.e., (1) no slip trial: the dummy-sheet-trial, (2) slip trial without fall, and (3) slip trial with fall in the slip-sheet-trials. The spatiotemporal relation between the dCOP and mCOP as well as dCOP-mCOP was compared among the three groups. The maximum value of dCOP-mCOP in *x*- and *y-*directions during the period from the right foot contacting on the 5^th^ force plate to the following left foot contacting on the 6^th^ force plate, i.e. during turning, was obtained. When a fall occurred, the maximum dCOP-mCOP in *x*- and *y-*directions was collected from the right foot contact on the 5^th^ force plate to the moment at which the load-cell force exceeded 30% of body weight.

### Statistical analysis

Statistical analyses were performed using SPSS (version 19.0, Chicago, IL, USA). As a result of a test for equality of valiance of the maximum dCOP-mCOP values in *x*- and *y-*directions among trials (no-slip, slip without fall, and fall), nonparametric Kruskal-Wallis test was performed to test if the maximum dCOP-mCOP in *x*- and *y-*directions was significantly different among trials (no-slip, slip without fall, and fall). Post hoc Mann-Whitney test was used to compare the maximum dCOP-mCOP among the conditions with modifying p-values using Bonferroni correction. Receiver operating characteristic (ROC) analysis [26] was also performed to determine the threshold value of the maximum dCOP-mCOP for fall occurrence. The significance level was set at 0.05.

## Results

### Spatiotemporal relation between dCOP and mCOP

Because of misstepping on the sheet during turning, the number of trials used in the analysis resulted in 22 for the dummy-sheet-trials and 55 for the slip-sheet-trials. Fifteen trials among 55 were identified as fall trials in the slip-sheet-trials.

**Fig 3:**
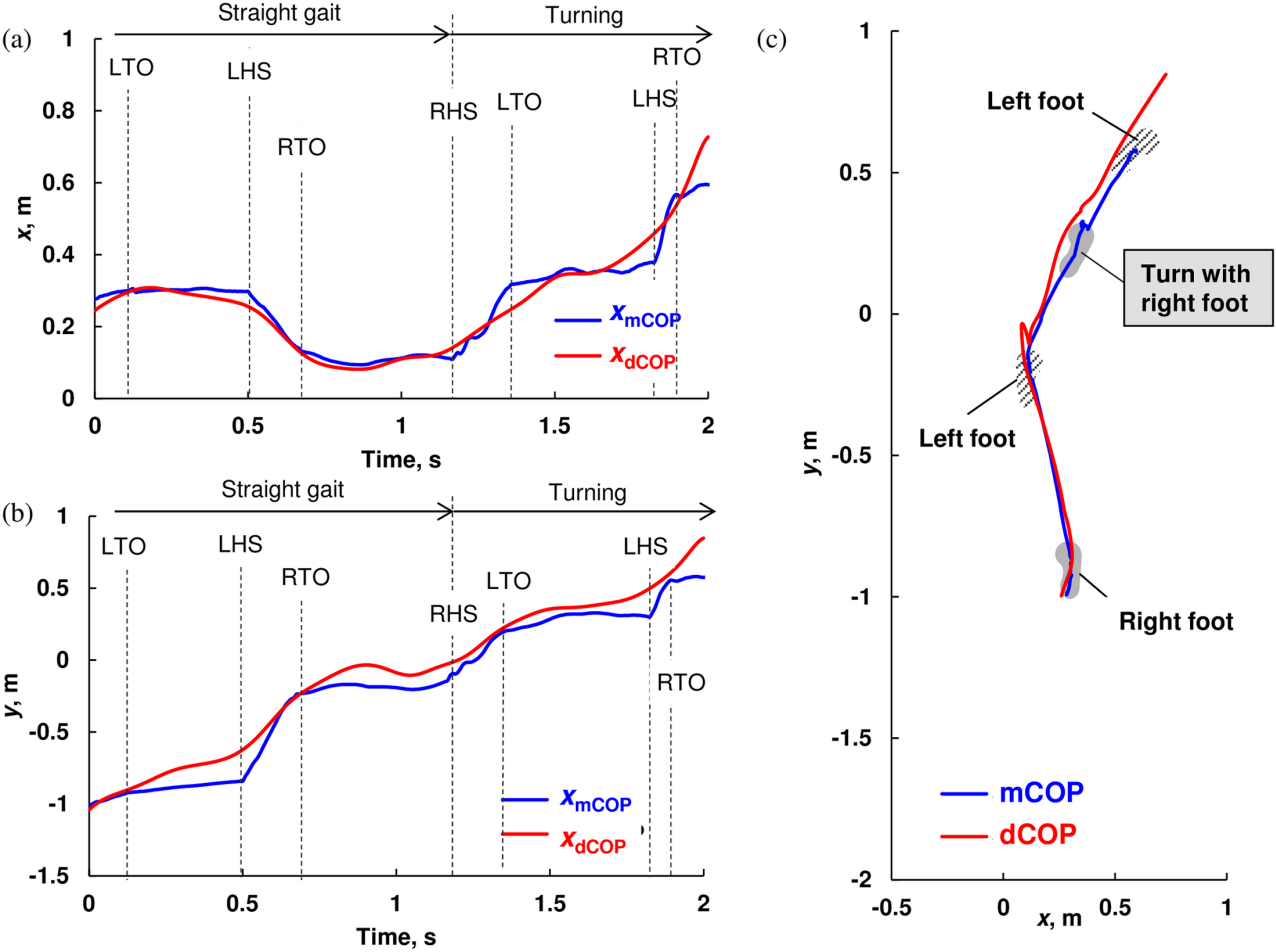
Representative trajectory of measured center of pressure (mCOP) and desired COP (dCOP) for straight walk and spin turn without slip on the dummy sheet in (a) *x*-direction, (b) *y*-direction, and (c) *x*-*y* plane. TO and HS mean toe-off and heel-strike, respectively.

**Fig 4:**
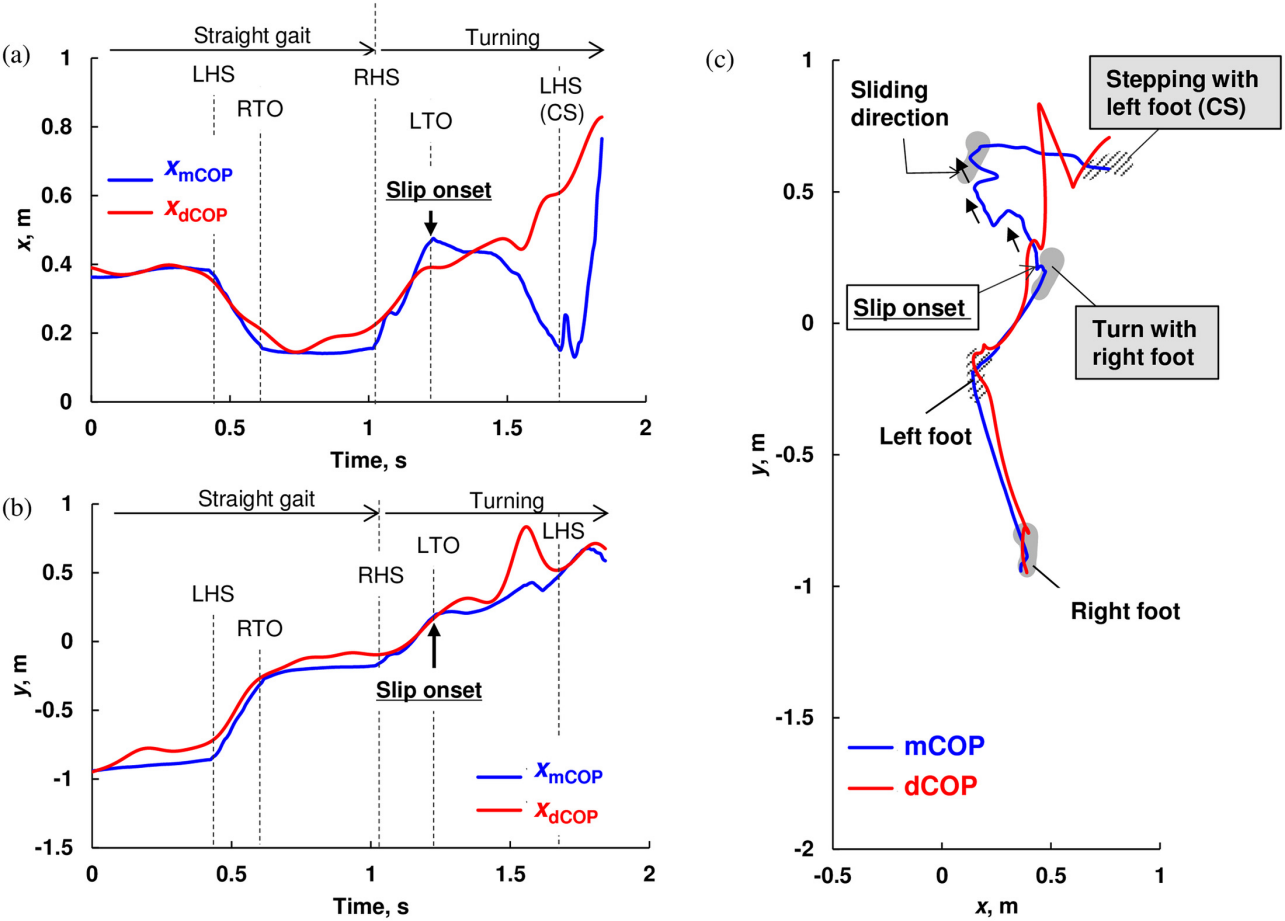
Representative trajectory of measured center of pressure (mCOP) and desired COP (dCOP) for straight walk and spin turn on the slip sheet with slip without fall in (a) *x*-direction, (b) *y*-direction, and (c) *x*-*y* plane. Slip on the right foot occurred during turning; however, subject successfully recovered postural balance due to stepping with the left foot. TO and HS mean toe-off and heel-strike, respectively. CS means compensatory step.

**Fig 5:**
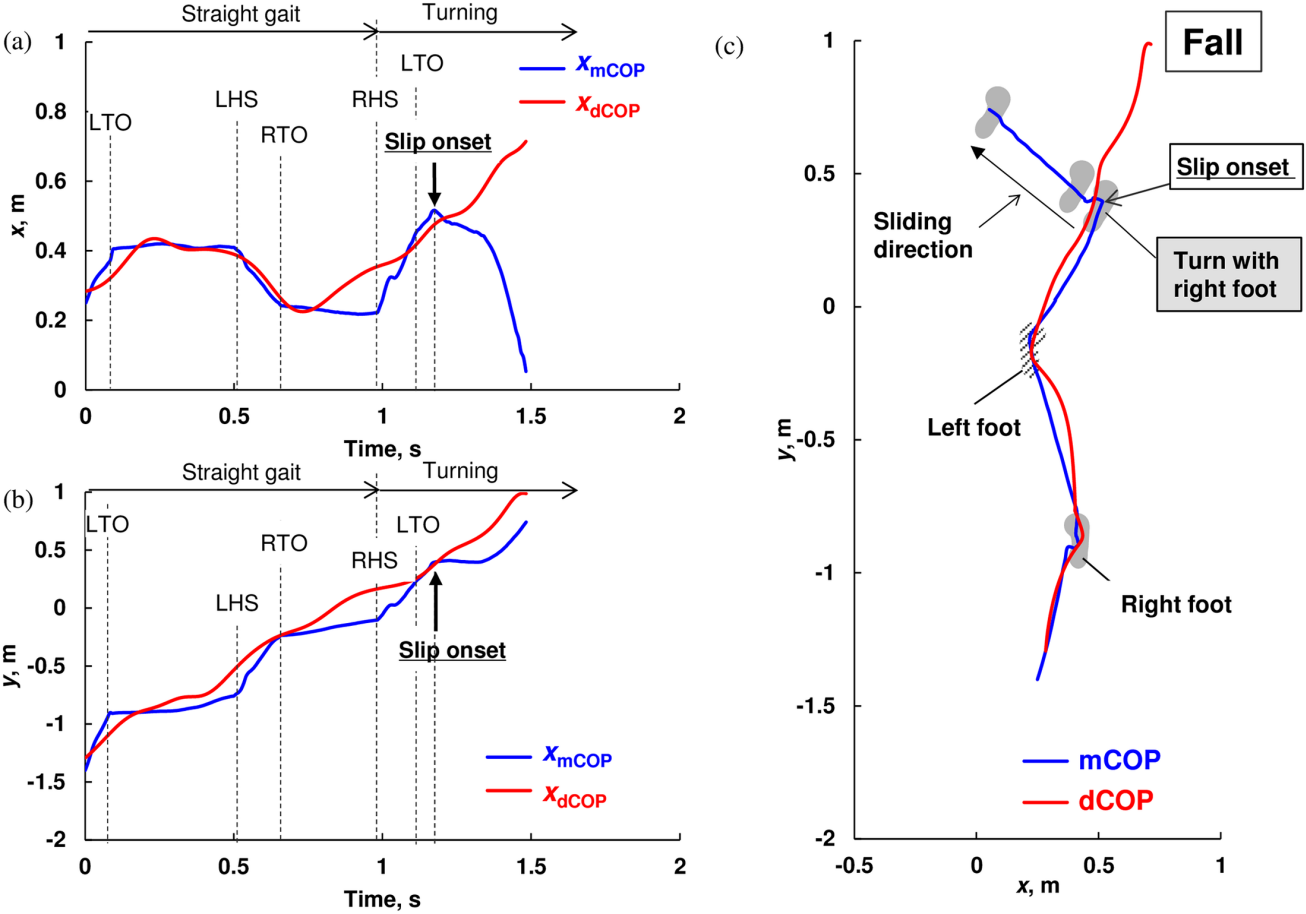
Representative trajectory of measured center of pressure (mCOP) and desired COP (dCOP) for straight walking and spin turn on the slip sheet with fall in (a) *x*-direction, (b) *y*-direction, and (c) *x*-*y* plane; slip on the right foot occurred during turning, and then subject could not step with left foot and finally fell. TO and HS mean toe-off and heel-strike, respectively.

Figs 3, 4 and 5 show the representative temporal and spatiotemporal trajectories of mCOP and dCOP for the no-slip trial during a dummy-sheet-trial, slip trial without fall during a slip-sheet-trial, and fall trial during a slip-sheet-trial, respectively. Note that *x*- and *y*-coordinates respectively represent the anteroposterior and mediolateral directions during straight walk, while they do not after turning. As shown in Fig 3, in no-slip trial, the subject turned with the right foot on the dummy-sheet, and successfully landed the left foot in the turning direction following the crossing over the right leg. As there was no slip, the misalignment of the dCOP and mCOP was relatively small during whole trial session, while the dCOP tended to locate anterior to the mCOP through straight gait and turning except at around the toe-off period (Fig 3(a) and 3(b)). This location of dCOP anterior to the mCOP during these periods indicates the required moment to propel the COM forward.

As shown in Fig 4, in the slip trial without fall, the subject initiated to turn with the right foot, which slipped, while he made a successful step by his left foot and continued to walk. The misalignment of the dCOP and mCOP after slip was larger than that in the no-slip trial (Figs 3 and 4). After slip, the mCOP of right foot moved in the left-forward direction and the dCOP moved in the right-forward direction, i.e. turning direction, which resulted in the separation of the dCOP and mCOP, mainly in *x*-direction (Fig 4(c)). However, then, the mCOP returned close to the dCOP owing to the compensating step by the left foot, resulting in postural recovery and continuing gait (Fig 4(c)). In this case, the subject successfully took a cross-over stepping.

On the other hand, as shown in Fig 5, in the slip trial with fall, the subject initiated to turn with the right foot, which slipped, and he was not able to make a successful step by the left foot as the slip was too large. In Fig 5, the right foot slides left-forward direction but the dCOP travels in the right-forward direction and the mCOP was not able to return close to the dCOP location.

### Maximum dCOP-mCOP in *x*- and *y*-directions

**Fig 6:**
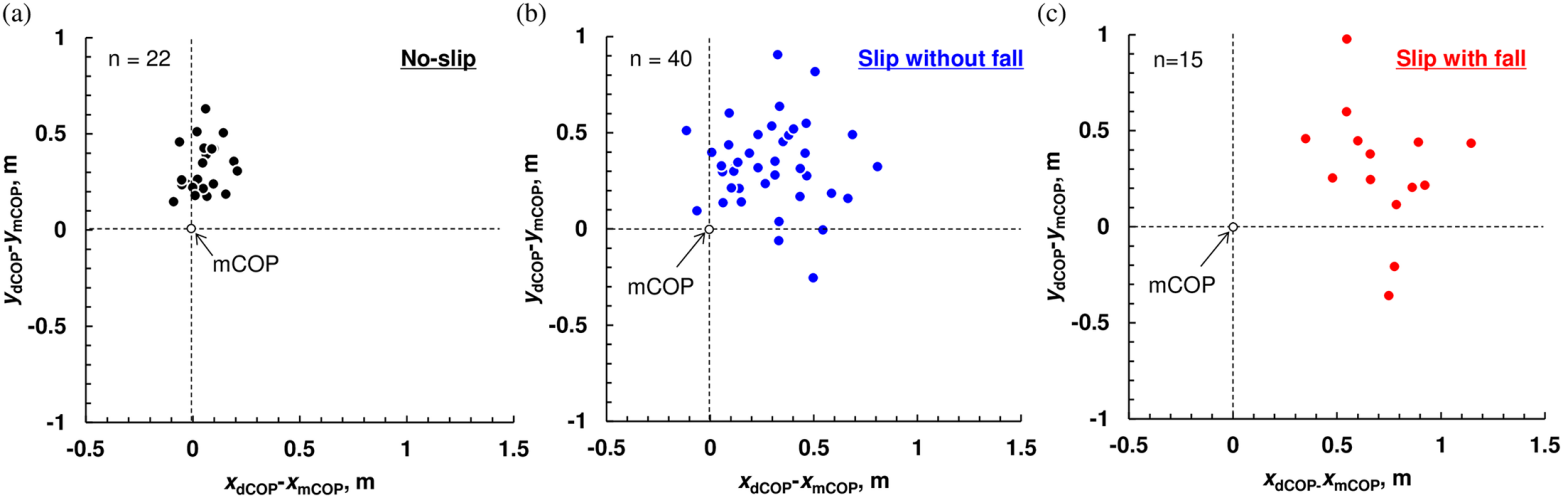
Relative location of dCOP with respect to mCOP for spin turn trials (a) on the dummy sheet (without slipping), (b) on the slip sheet with slip but not fall, and (c) on the slip sheet with fall at which the dCOP–mCOP took the largest value during turning in each trial. Original point corresponds to the location of mCOP, and each plot indicates dCOP location.

Fig 6 shows the location of the dCOP with respect to the mCOP (set as the origin of the coordinate) when the dCOP-mCOP took the largest value during turning in each trial. In the no-slip trials (Fig 6(a)), the dCOP tends to locate ahead of the mCOP on *x*-*y* plane whereas the dCOP dominantly locates on the right side of the mCOP on *x*-*y* plane in the slip trials without fall (Fig 6(b)). The dCOP locates farther on the right side of the mCOP on *x*-*y* plane when a fall occurred in the slip trials (Fig 6(c)).

**Fig 7:**
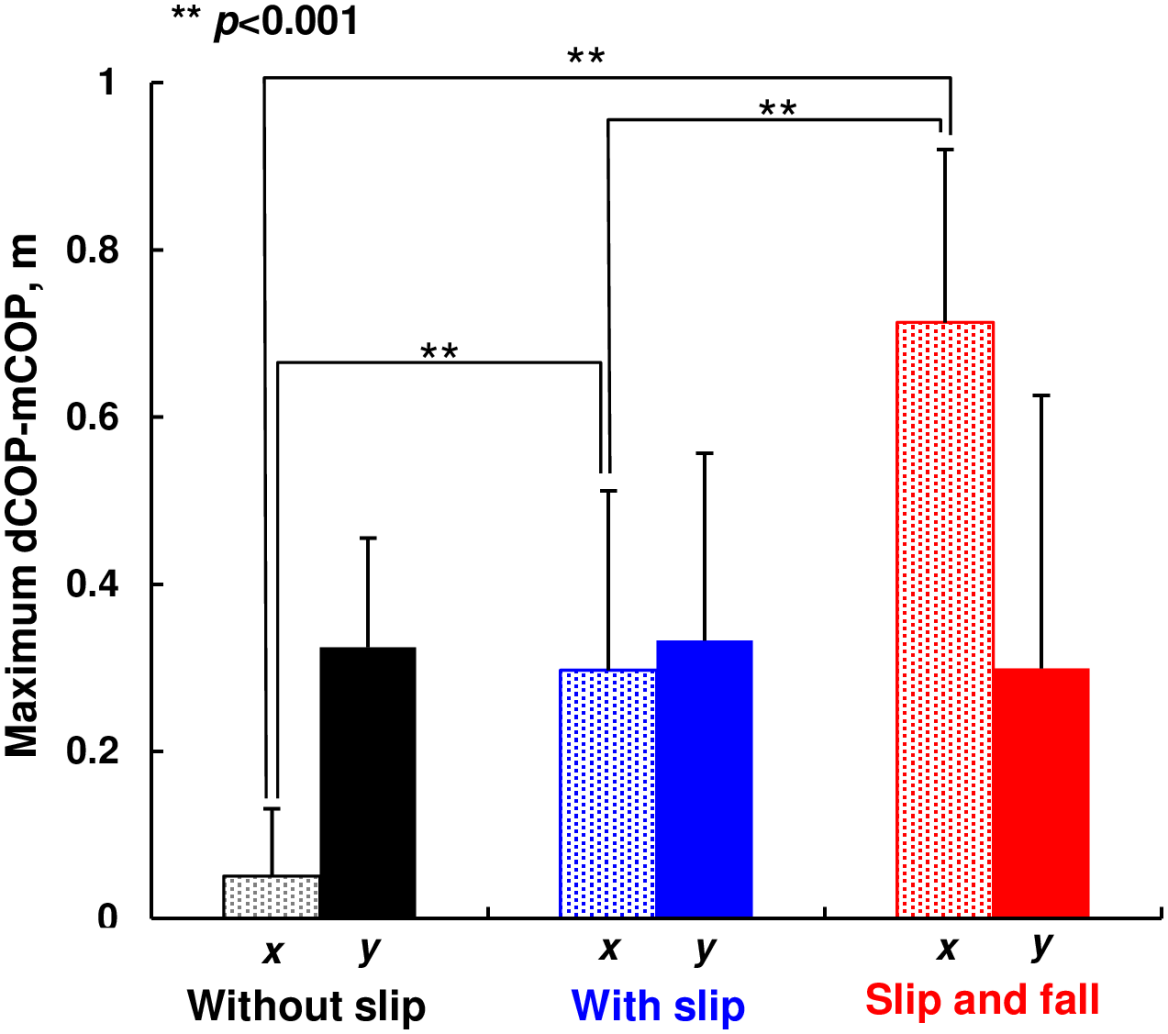
Mean value of the maximum dCOP-mCOP in *x*- and *y*-directions during spin turn.

Fig 7 shows mean values of the maximum dCOP-mCOP in the *x*- and *y*-directions for each condition. Kruskal-Wallis test indicated that the maximum dCOP-mCOP in *y*-direction was not significantly different among the result of trials (no-slip, slip without fall, slip with fall) (*p* = 0.064). On the contrary, the maximum dCOP-mCOP in *x*-direction was significantly different among the result of trials (*p* < 0.001). Post hoc Mann-Whitney test revealed that the maximum dCOP-mCOP in *x*-direction with slip without fall was significantly larger than that with no slip (*p* <0.001), and the maximum dCOP-mCOP in *x*-direction with fall was significantly larger than that with no-fall after slip (*p*<0.001). Note that the same results were obtained for the maximum dCOP-mCOP normalized by the subject’s body height, which we did not show here.

### Threshold of maximum dCOP-mCOP in *x*-direction for fall followed by slip

**Fig 8:**
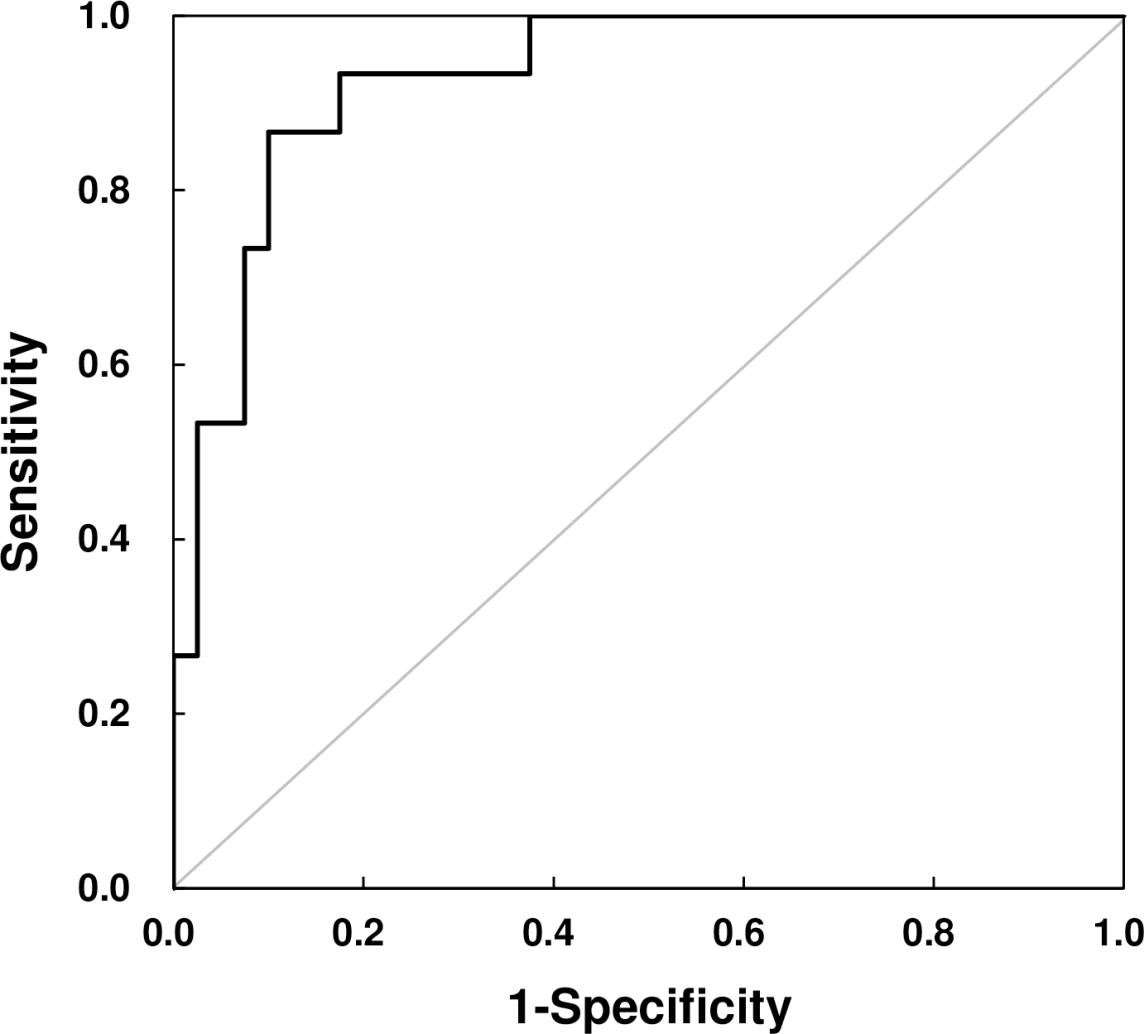
ROC curve for prediction of fall.

Fig 8 shows ROC curve for the maximum dCOP-mCOP in *x*-direction. The horizontal (sensitivity) and vertical (1-specificity) axes mean the proportion of trials without falling and that of trials with fall, respectively. The area under the curve (AUC) is a reflection of how the maximum dCOP-mCOP in *x*-direction is at distinguishing with trials without fall and those with fall. The AUC greater than 0.9 has high accuracy, while 0.7–0.9 indicates moderate accuracy, 0.5–0.7, low accuracy [26]. The AUC was 0.93 (*p* < 0.001), indicating that the maximum dCOP-mCOP in *x*-direction accurately predicts fall [AUC >0.9 [26]]. The threshold or the optimal cutpoint of the dCOP-mCOP in *x*-direction was determined using the point on the ROC curve closest to the (0, 1) point [23]. The threshold of the dCOP-mCOP in *x*-direction for distinguishing at-risk of fall from that at no-risk of fall was 0.55 m (sensitivity: 0.867; 1-specificity: 0.100). Note that the ROC analysis also indicated that the maximum dCOP-mCOP (resultant distance of the dCOP-mCOP in *x*- and *y*-direction) was a good indicator of fall due to slip during turning (AUC = 0.91, *p* < 0.001); more specifically, the maximum dCOP-mCOP in *x*-direction was slightly better.

## Discussion

We presented that the subject was not able to continue gait, i.e. fell, when the dCOP-mCOP reached a certain value, particularly in *x*-direction (Figs 7 and 8). When slip occurred, mCOP moved toward left-forward direction on *x-y* plane while dCOP was keeping moving right-forward on *x-y* plane resulting in a large deviation between mCOP and dCOP, especially in *x*-direction. In this case, subject should control the swing left foot in *x*-direction to step over the dCOP position in order to locate the global mCOP (at double support period) close to the following dCOP location, which can reduce the moment around COM, leading to a successful recover of balance. The large dCOP-mCOP in *x*-direction caused a large moment around the COM in *x*-direction. When this moment became too large, the subject was not able to make a successful compensatory step. This threshold value of dCOP-mCOP was 0.55 m revealed by ROC analysis (Fig 8). On the contrary, the maximum dCOP-mCOP in *y*-direction was not different among conditions (Fig 7). The maximum dCOP-mCOP in *y*-direction was almost constant regardless of occurrence of slip, since mCOP moved left-forward while dCOP moved right-forward in *x-y* plane as abovementioned. Although the moment induced by dCOP-mCOP does not directly propel the body but only a horizontal force propels the body, this value of the maximum dCOP-mCOP in *y*-direction probably indicates a necessary magnitude of forward moment to propel the COM.

The margin of stability [2, 27] indicates whether the posture is stable or unstable and there are uncertainly about what this measure has to offer when the XCoM is outside the BOS [13]. Although the instantaneous COM stability can identify fall and recovery from forward slipping during straight walking better than the other stability measures [4, 6], there is a practical difficulty in using this in a complex and realistic situation such as slip during turning gait. Furthermore, both measures need BOS measurement. On the other hand, the dCOP-mCOP increases with an increase of the moment around the COM, which can represent a loss of postural stability and a difficulty in making successful compensatory stepping. As shown in Fig 4(a), the dCOP was located outside the BOS boundary in *x*-direction after slip because the dCOP-mCOP was significantly greater than the BOS width (i.e., width of the shoe: approximately 0.1 m); however, the subject successfully recovered his balance by making compensatory stepping as the resultant discrepancy between dCOP and mCOP was relatively small. On the other hand, although the dCOP was also located outside BOS boundary in *x*-direction after slip (Fig 5(a)), the resultant discrepancy between dCOP and mCOP was larger resulting in falling without being able to make compensatory step. Thus, dCOP can be used even when it is outside of the BOS and can continuously indicate the dynamic postural stability. This means that there is no need to measure the BOS boundary in assessing the dynamic postural stability based on the dCOP concept whereas other measures need BOS boundary measurement. We believe that our dCOP concept can be used as more global measure of dynamic postural stability at the actual daily situation with a large perturbation due to such as induced-slip, which needs compensatory stepping to recover balance. Future studies are required to generalize the applicability of dCOP concept.

The results of this study evidently showed that there is a threshold of the maximum dCOP-mCOP in *x*-direction to determine if slip-induced fall occurs during turning or not. As the threshold value must be affected by the ability in controlling stepping, aging and/or physical impairment can significantly influence on the threshold value. A fall is a major cause of life threating accidents for elderly people [28, 29] and a slip-induced fall has been suggested as a source of large percentage of the total fall-related accidents among the elderly [30–32]. The slipperiness during walking is characterized by the ratio between horizontal ground reaction force and vertical ground reaction force, which was referred to as “required coefficient of friction (RCOF)” [33]. RCOF is considered as the minimum coefficient of friction to prevent slip during walking. The elderly generally walk slower with slower heel contact velocity, thus, resulting in lower RCOF (i.e. friction demand) than the young [34]. This indicates that the elderly are less likely to slip compared to the young. Therefore, the reason that could account for the high risk of slip-induced fall in the elderly is the lack of the ability in balance recovery by compensatory stepping after slip. That is, the elderly often appear to experience difficulty when stepping in response to lateral perturbation [14, 35–37]. From the perspective of this study, the elderly may have the high risk of fall, because they can tolerate only a shorter dCOP-mCOP in *x*-direction than the one for the young (0.55 m in this study) due to the lack of ability in balance recovery by stepping. Future studies are required to investigate this hypothesis.

Since the dCOP-mCOP is related to the magnitude of moment around COM, our dCOP concept may be applicable to the gait variability, which is significantly related to fall probability of the elderly [38, 39] or patients with movement disorders such as stroke and Perkinson’s disease [40–42]. The misalignment of dCOP and mCOP may provide rationale behind the variability of gait parameters such as step length, step width of the elderly or patients with movement disorders. The dCOP may also yield insight into the desired foot placement for stable gait and can be utilized in rehabilitation field.

As shown in Fig 6, the dCOP-mCOP in *y*-direction is systematically positive, which means that the dCOP overall locates anterior to the mCOP. Similarly, Hof [43] indicated that stable forward locomotion needs “constant offset control” which is the stable control of COP position made by positioning the COP at a constant distance behind the XCoM. These must be related to the required moment to propel the COM forward. Further research is needed to investigate this point with another experimental set-up using such as slipping trial only in *y*-direction or trip during walking.

Our inverted pendulum model assumes that the whole body is rigid. On the contrary, Hof [44] developed a standing multi-segment human model that contains multiple rigid segments connected at joints, which could remove the restriction of “rigid whole-body” in our model. Comparing with Hof’s model, the dCOP-mCOP corresponds to *x*_h_ in his model, which is the derivative of the total angular moment with respect to the COM divided by the vertical ground reaction force; however, no balance issue has been addressed by use of *x*_h_ yet. In future work, it would be beneficial to incorporate Hof’s model into ours to generalize the dCOP concept.

A potential limitation of this study was that our experimental design could not eliminate the possibility of anticipation effects on the subject’s reactive responses. Therefore, our task is close to a situation when a man carefully walks on ice. However, this does not affect our conclusion as we investigated the mechanical consequence of slips. Regardless of the subject’s cautiousness and anticipation, all subjects slipped when dCOP-mCOP became more than a threshold value, which is the mechanical consequence. Another limitation of this study may be that the participants were all young adult males. Further studies are needed to investigate whether the dCOP concept is applicable to slip and fall of elderly and both genders. Normalization of the magnitude of dCOP-mCOP with respect to subject’s height or lower limb, etc. also is needed to extend subject gender and age. Additionally, although, turning gait was conducted in this study, (1) as slip and fall are more likely during turning than walking straight [15,17] and (2) to confirm the feasibility of our method under trial with a large perturbation, i.e. the situation in which the COM is apparently outside, which cannot be realized by straight walk, we need to confirm if this dCOP concept can be used in slip and fall during turning with other turning angles and other types of gait such as straight gait, gait initiation and gait termination. The present study is the first attempt to use the dCOP-mCOP as a measure for dynamic balance evaluation. It is necessary to compare this measure with the other measures such as the COM stability, the margin of stability, etc., in future work.

In summary, the current study demonstrated that it becomes difficult to recover balance of COM and to continue gait, when the misalignment of the dCOP and mCOP (dCOP-mCOP) in *x*-direction increases up to a certain value (0.55 m) due to a large perturbation such as slips during turning. The results suggest that the dCOP-mCOP distance in *x*-direction is good indicator of falling. These results indicate the feasibility of the dCOP concept in assessing the risk of fall due to induced slip during turning.

## Supporting Information

S1 Table The maximum values of dCOP-mCOP in the *x*- and *y*-directions. (XLSX)
